# Characteristics of complete chloroplast genome of a high-quality forage on Qinghai-Tibet Plateau, *Medicago archiducis-nicolai* Sirj. (Fabaceae：Trifolieae)

**DOI:** 10.1080/23802359.2020.1861558

**Published:** 2021-01-19

**Authors:** Jiuxiang Xie, Chengbo Liang, Zongren Li, Zhongke Ji, Weiyou Ou, Hua Liu, Haichun Wang, Kazhuocairang, Zhiqiang Hou

**Affiliations:** a State Key Laboratory of Plateau Ecology and Agriculture, College of Agriculture and Animal Husbandry, Qinghai University, Xining, China; b Forest Station of Youganning Town, Henan Mongolian Autonomous County, Youganning, China; c Qinghai Provincial General Grassland Station, Xining, China; d Grassland comprehensive professional team of Henan Mongolian Autonomous County, Youganning, China

**Keywords:** *Medicago archiducis-nicolai*, chloroplast genome, Fabaceae, phylogenetic analysis

## Abstract

*Medicago archiducis-nicolai* Sirj. is a well-known high-quality forage as its good palatability and strong tolerance to drought, cold and saline-alkali stress. Here, the complete chloroplast genome sequence of *M. archiducis-nicolai* was reported. The size of the complete chloroplast genome is 127,072 bp in length. The chloroplast genome has no inverted repeat (IR) regions, which is very common in the family Fabaceae. The *M. archiducis-nicolai* chloroplast genome encodes 106 genes: 72 protein-coding genes, 30 tRNAs, and 4 rRNAs. The phylogenetic analysis result strongly suggested that *M. archiducis-nicolai* is a distinct lineage in *Medicago*, being sister to highly supported clade composed of three species (*M. hybrida, M. papillosa* and *M. sativa*).

*Medicago archiducis-nicolai* Sirj. is a perennial plant of Medicago, which is one of the core species of Section Pialcarpae (Small and Marcel 1989). *Medicago archiducis-nicolai* is mainly distributed in Qinghai, Tibet, and adjacent to high-altitude areas. It is the only perennial wild alfalfa that can survive the winter in the natural alpine grassland of Qinghai Tibet Plateau (Jin et al. 2013). It is widely distributed in the areas with an altitude of 1500–4250m (Li et al. 1997; De and Xu 2009), and grows better in the sunny slope grassland, river beach and secondary bare land. It has strong tolerance to drought, cold and saline-alkali stress (Balabaev 1934). Compared with *Medicago ruthenica*, *M. archiducis-nicolai* as an indigenous species in the Tibetan Plateau, has a more severe growth environment and stronger adaptability to extreme environments (Wang et al. 2020). At the same time, *M. archiducis-nicolai* is a perennial wild legume species, has high crude protein content and good palatability (Dekejia and Xu 2009) has dense underground rhizomes with rich nodules, and also has strong barren and trampling resistance (Liu 1987; Li 2007a). For these reasons, *M. archiducis-nicolai* is considered to be a high-quality perennial leguminous forage resource with domestication potential, which is expected to be cultivated and utilized in areas where *M. sativa* and other alfalfa species cannot survive the winter (Li 2007b). However, no studies on the plastome of *M. archiducis-nicolai* have been published. In this study, the complete chloroplast genome of *M. archiducis-nicolai* (Genbank accession number: MN901634) was sequenced on the Illumina NovaSeq Platform (Illumina, San Diego, CA, USA), which will provide genetic and genomic information to promote its ecological restoration and systematics research of Fabaceae. The raw data was submitted on Sequence Read Archive (Number: SRR12951164).

In this study, *M. archiducis-nicolai* were collected from Garang village, Guide County, Haidong City, Qinghai Province, China (36.32°N, 101.52°E). The fresh and young leaves were dried immediately by silica gels. The complete chloroplast genome of *M. archiducis-nicolai* was extracted from the dried leaves (about 0.2 g) with a modified CTAB method. The voucher specimen was kept in the Herbarium of the Northwest Institute of Plateau Biology, Chinese Academy of Sciences (HNWP, XIE2019014). Genome sequencing was performed using the Illumina NovaSeq Platform at Genepioneer Biotechnologies Inc., Nanjing, China. The trimmed reads were mainly assembled by SPAdes (Bankevich et al. 2012). Then, PCGs, rRNAs and tRNAs were annotated by blast v2.2.25, hmmer v3.1b2 and aragorn v1.2.38, respectively.

The complete chloroplast genome of *M. archiducis-nicolai* has an atypical chloroplast genome structure with a length of 127,072 bp. This chloroplast genome has no inverted repeat (IR) regions, which is very common in the family Fabaceae. The GC content of the whole chloroplast genome is 34.23%. A total of 106 functional genes were annotated, including 72 protein-coding genes (PCGs), 30 tRNA genes and 4 rRNA genes. 14 of them contain 1 intron and 1 of them contains 2 introns.

Eight complete chloroplast genomes of Fabaceae (the number from Medicago and Pisum are 7 and 1, respectively) and two outgroups (two species from Crassulaceae Phedimus) were used for constructing maximum likelihood with 1000 bootstrap repeats (model: K3Pu + F) by W-IQ-TREE (Trifinopoulos et al. 2016) after aligned by MAFFT 7 (Katoh and Standley 2013) (Figure 1). The phylogenetic tree showed that *M. archiducis-nicolai* was sister to the clade composed of three species (*M. hybrida, M. papillosa and M. sativa*).

**Figure 1.**
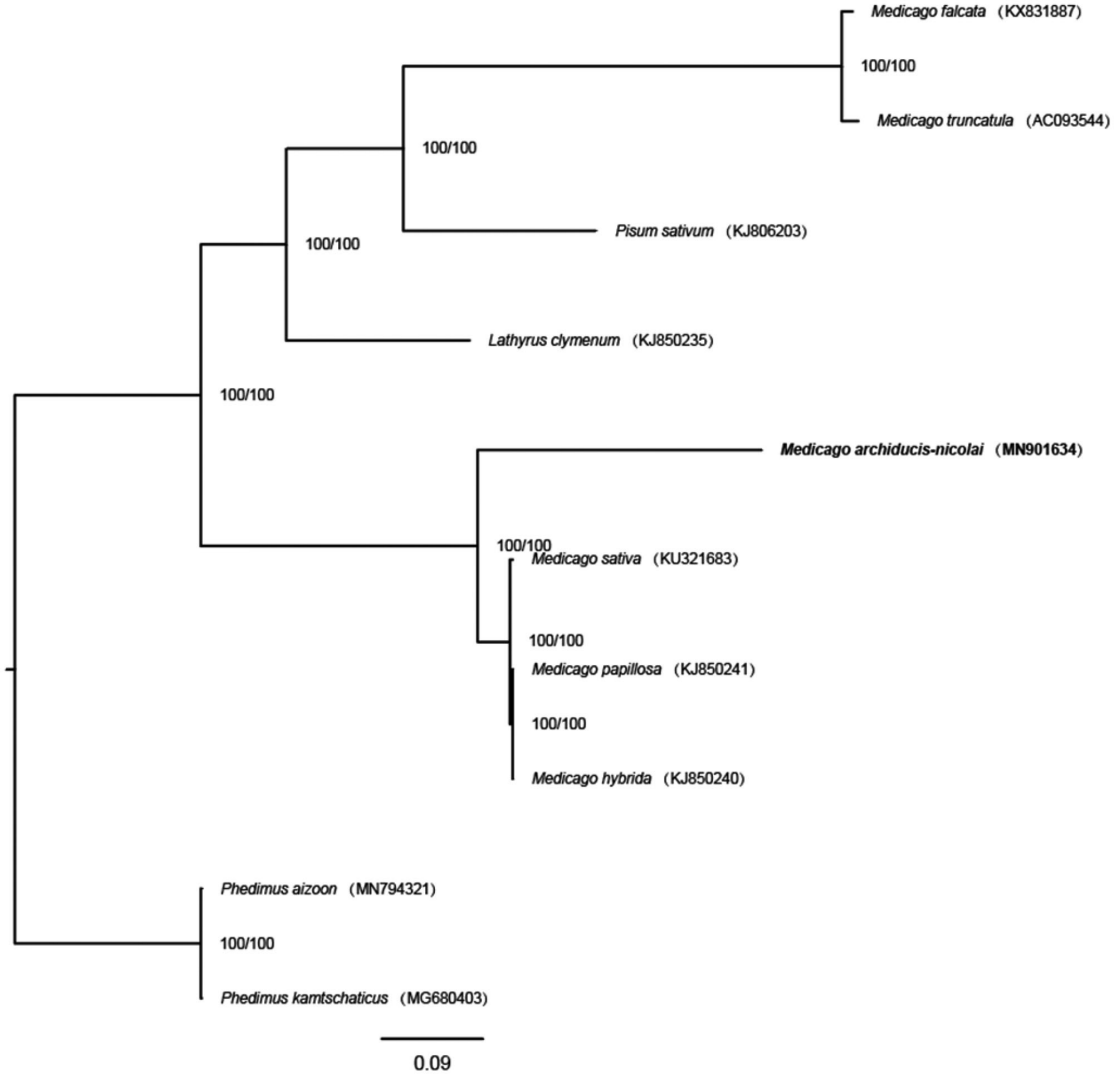
The maximum likelihood tree based on 10 complete chloroplast genome sequences. Support values written on the branches: SH-aLRT support (%)/ultrafast bootstrap support (%).

## Data Availability

The data that support the findings of this study are openly available in GenBank of National Center for Biotechnology Information at https://www.ncbi.nlm.nih.gov (Reference number: MN901634) and Sequence Read Archive at https://www.ncbi.nlm.nih.gov/search/all/?term=SRR12951164 (Number: SRR12951164).
